# X-ray spectroscopic study of chemical state in uranium carbides

**DOI:** 10.1107/S160057752101314X

**Published:** 2022-01-27

**Authors:** Sergei M. Butorin, Stephen Bauters, Lucia Amidani, Aaron Beck, Stephan Weiss, Tonya Vitova, Olivier Tougait

**Affiliations:** a Condensed Matter Physics of Energy Materials, X-ray Photon Science, Department of Physics and Astronomy, Uppsala University, PO Box 516, SE-751 20 Uppsala, Sweden; b Helmholtz-Zentrum Dresden-Rossendorf (HZDR), Institute of Resource Ecology, PO Box 510119, 01314 Dresden, Germany; c The Rossendorf Beamline at ESRF – The European Synchrotron, CS40220, 38043 Grenoble Cedex 9, France; d Institute for Nuclear Waste Disposal (INE), Karlsruhe Institute of Technology, PO 3640, D-76021 Karlsruhe, Germany; e Univ. Lille, CNRS, Centrale Lille, Univ. Artois, UMR 8181 – UCCS – Unité de Catalyse et Chimie du Solide, F-59000 Lille, France

**Keywords:** uranium carbides, X-ray spectroscopy, chemical state

## Abstract

UC and UMeC_2_ (Me = Fe, Zr, Mo) carbides were studied by the high-energy-resolution fluorescence-detected X-ray absorption (HERFD-XAS) technique at the U *M*_4_ and *L*_3_ edges. Both U *M*_4_ and *L*_3_ HERFD-XAS reveal some differences between UMeC_2_ and UC; there are differences also between the *M*_4_ and *L*_3_ edge results for both types of carbide in terms of the spectral width and energy position. The observed differences are attributed to the consequences of the U 5*f*, 6*d*–4*d*(3*d*) hybridization in UMeC_2_. Calculations of the U *M*_4_ HERFD-XAS spectra were also performed using the Anderson impurity model (AIM). Based on the analysis of the data, the 5*f* occupancy in the ground state of UC was estimated to be 3.05 electrons. This finding is also supported by the analysis of U *N*_4,5_ XAS of UC and by the results of the AIM calculations of the U 4*f* X-ray photoelectron spectrum of UC.

## Abstract 2

UC and UMeC_2_ (Me = Fe, Zr, Mo) carbides were studied by the high-energy-resolution fluorescence detected X-ray absorption technique at the U *M*_4_ and *L*_3_ edges. The data were analyzed using the Anderson impurity model.

## 1. Introduction

Mixed carbides (U, Pu)C are considered as promising fuels for the generation-IV nuclear reactors. Compared with standard mixed oxide fuel (U, Pu)O_2_, mixed carbides are expected to improve reactor performance due to higher thermal conductivity and higher metal atom density, better structural stability compared with standard nuclear fuels and better chemical compatibility with fuel cladding materials, such as stainless steel. However, actinide carbides are much less studied compared with actinide oxides. Therefore, the choice of this fuel for generation-IV reactors requires a significant amount of research to be done from both the fundamental and applied points of view. In particular, better understanding of the electronic structure will improve knowledge relevant for evaluating the technological performance of actinide systems. For example, it is important to gain insight into the cation charge distribution and the carbon/metal (C/*M*) ratio in carbide materials as these are key parameters for assessing thermodynamic, chemical and physical properties of the systems in question.

An important physical quantity in this respect is the number of electrons in the actinide 5*f* shell which is linked to the actinide chemical state, cation charge distribution and (non)stoichiometry, and affects important properties, such as *e.g.* thermal conductivity. The *ab initio* calculations, based on the density functional theory (DFT) formalism which takes into account the Coulomb interaction between the 5*f* electrons (DFT + *U*) or based on dynamical mean-field theory (DFT + DMFT), provide information about the electronic structure of actinide systems, but some difficulties remain in estimating accurately the occupancy of the 5*f* shell in compounds.

X-ray spectroscopy is known to be an efficient tool to probe the electronic structure and helps to extract information about the 5*f* occupancy during analysis of the spectra within the Anderson impurity model (AIM) (Anderson, 1961 ▸). Here we apply the advanced X-ray spectroscopic technique, high-energy-resolution fluorescence-detection X-ray absorption spectroscopy (HERFD-XAS), which significantly improves the experimental resolution and therefore enhances the sensitivity of the method to changes in the chemical state of actinides (Kvashnina *et al.*, 2013 ▸).

For UC, research groups which measured X-ray photoemission (XPS) spectra (Erbudak & Keller, 1979 ▸; Ishii, 1993 ▸; Ejima *et al.*, 1993 ▸; Ito *et al.*, 2001 ▸; Eckle *et al.*, 2004 ▸), probing the valence band, and inverse photoemission spectra (Ejima *et al.*, 1993 ▸), probing the conduction band, compared these spectra with conventional band structure calculations. By making such a comparison, they suggested that the states in UC have a completely itinerant character. As a consequence, UC is often compared to U metal in terms of the electronic structure character (Eckle *et al.*, 2004 ▸; Vigier *et al.*, 2008 ▸). However, the results of advanced DFT + *U* (Shi *et al.*, 2009 ▸; Ducher *et al.*, 2011 ▸; Wdowik *et al.*, 2016 ▸), DFT + DMFT (Yin *et al.*, 2011 ▸) and hybrid DFT (Wen *et al.*, 2013 ▸) calculations suggest partial localization of the U 5*f* states and indicate that the electron correlation effects are certainly not negligible in UC by deriving the appreciable value for the 5*f*–5*f* Coulomb interaction (*U*_*ff*_). However, no consensus on the *U*_*ff*_ strength has been established because the estimated values varied between 2 and 5 eV. The significance of the electron correlation effects for the 5*f* shell is also supported by the AIM analysis of the core-level 4*f* XPS spectra even for intermetallic U systems (Okada, 1999 ▸).

We report HERFD-XAS measurements at the U *M*_4_ edge of UC and their analysis within the framework of AIM. The latter allowed us to derive information about the ground state and to estimate the 5*f* occupancy. The obtained results were further supported by the analysis of the U 4*d* XAS (*N*_4,5_ edges) and available U 4*f* XPS data for UC. The U *M*_4_ and *L*_3_ HERFD-XAS data of UC were also compared with HERFD-XAS spectra of UMeC_2_, where Me = Fe, Zr, Mo. These compounds also show metallic character and are of interest as products of the interaction between the uranium carbide fuel and cladding and canning materials. Furthermore, UMoC_2_ can exist as a fission product (Peniel *et al.*, 2012 ▸).

## 2. Experimental

The samples were prepared by arc melting the relevant proportions of metallic uranium (depleted uranium, Framatome, 99.9%) and graphite pieces (Mersen) with transition metals, such as iron, molybdenum and zirconium (Alfa-Aesar, 99.99%). To ensure a good homogeneity, the buttons were turned and re-melted four times. Each melting was performed under argon pressure of about 0.8 bar, after two vacuum/Ar purges.

The UFeC_2_ sample was annealed at 1200°C for 12 h in an induction furnace under argon. All samples were characterized by X-ray diffraction (XRD), using a Bruker D8 Advance diffractometer, with monochromatic Cu *K*α radiation. XRD patterns were refined by the Rietveld method, using the *FullProf* software (Rodríguez-Carvajal, 2001 ▸). The samples were also characterized using scanning electron microscopy (SEM), coupled to energy-dispersive spectroscopy (EDS). The SEM imagery using backscattered electrons gives a representation of the microstructure of the samples based on the compositional contrast between the different phases present. EDS analyses are also not suitable for estimating the concentration of carbon, due to its small number of electrons. These analyses are thus mainly used to evaluate the U/Me ratios when Me = Fe, Mo and Zr.

The chemical and structural characterizations revealed that UC, UMoC_2_ and UZrC_2_ form directly from solidification as pure samples and with their expected crystalline structure: UC, NaCl type, 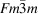 space group with *a* = 4.9560 (2) Å; UMoC_2_, UMoC_2_ type, *Pnma* space group with *a* = 5.6232 (1), *b* = 3.2485 (1) and *c* = 10.9935 (2) Å; and UZrC_2_, NaCl type, 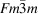 space group with *a* = 4.8312 (2) Å. The EDS analyses confirmed the U/Me ratio of 0.50 (2) for the UMeC_2_ compounds, Me = Mo and Zr.

The examination of the as-cast UFeC_2_ ingot found a multiphase sample containing UFeC_2_ but also UC and Fe_2_C in small portions. This sample became a single phase after annealing at 1200°C for 12 h. The X-ray diffractogram was fully indexed according to the ScCoC_2_ type, *P*4/*nmm* space group with *a* = 3.5237 (2) and *c* = 7.4200 (3) Å.

The measurements in the energy range of the U 3*d* and 2*p* X-ray absorption edges were carried out at the CAT-ACT beamline (Zimina *et al.*, 2017 ▸) of the KARA (Karlsruhe research accelerator) facility in Karlsruhe, Germany. The incident energies were selected using the 〈111〉 reflection from a double Si-crystal monochromator. The XAS scans were measured in the HERFD mode using the X-ray emission spectrometer (Zimina *et al.*, 2017 ▸). Only one crystal analyzer of the spectrometer was used in all the measurements. The sample, analyzer crystal and photon detector were arranged in a vertical Rowland geometry. The U HERFD spectra at the *M*_4_ (3*d*_3/2_ → 5*f*_5/2_, 7*p* transitions) edge were obtained by recording the outgoing photons with an energy corresponding to the maximum of the U *M*β (4*f*_5/2_ → 3*d*_3/2_ transitions) X-ray emission line, as a function of the incident energy, and the U HERFD at the *L*_3_ (2*p*_3/2_ → 6*d*, 7*s* transitions) edge was recorded at the maximum of the U *L*α (3*d*_5/2_ → 2*p*_3/2_ transitions) line. The right emission energy was selected using the spherically bent Si 〈220〉 crystal analyzer (with 1 m bending radius) aligned at 75° Bragg angle for the measurements at the U *M*_4_ edge and Ge 〈777〉 at 77° Bragg angle for the measurements at the U *L*_3_ edge. The HERFD data were recorded at one emission energy and the spectrometer was not moved between the scans. The spectral intensity was normalized to the incident flux. The total energy resolution was estimated to be ∼0.7 eV at the U *M*_4_ edge and ∼2.6 eV at the U *L*_3_ edge.

The measurements in the energy range of the U *N*_4,5_ (4*d* → 5*f*, 7*p* transitions) edges of UC were performed at beamline 5.3.1 of the MAXlab (Denecke *et al.*, 1999 ▸). U 4*d* XAS data were measured in the total electron yield (TEY) mode using drain current on the sample. The incidence angle of the incoming photons was close to 90° to the surface of the sample. The monochromator resolution was set to ∼400 meV at 740 eV during measurements at the U 4*d* edges.

Samples were prepared and sealed in a special argon-filled container at the licensed laboratory of HZDR and were transported to KARA under inert conditions. All samples were mounted in the form of pellet pieces within triple holders with an 8 µm Kapton window on the front side, serving as first confinement. Three of such holders were mounted in one larger cell, with a 13 µm Kapton window on the front side. The second confinement chamber was constantly flushed with He. The entire spectrometer environment was contained within a He box to improve signal statistics.

## 3. Computational details

The AIM (Anderson, 1961 ▸) was used for the calculations which included the 5*f* and core 3*d*(4*d*) or 4*f* states on a single actinide ion and the valence-band states. The calculations were performed in a manner described in the literature (Butorin *et al.*, 1996 ▸; Nakazawa & Kotani, 2002 ▸; Okada, 1999 ▸).

The total Hamiltonian of a system can be written as 

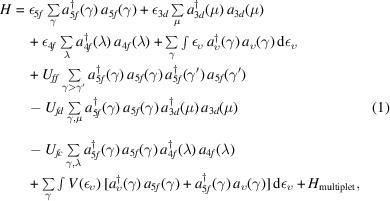

where ε_5*f*_, ε_3*d*_, ε_4*f*_ and ε_υ_ are one-electron energies of actinide 5*f*, core 3*d* and 4*f* levels and valence band, respectively, and 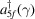 , 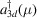 , 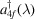 , 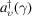 are electron creation operators at these levels with combined indexes γ, μ and λ to represent the spin and orbital states of the 5*f*, 3*d* and 4*f* and valence-band electrons. *U*_*ff*_ denotes the 5*f*–5*f* Coulomb interaction, *U*_*fd*_ and *U*_*fc*_ are the 3*d* and 4*f* core hole potentials, respectively, acting on the 5*f* electron. *V*(ε_υ_) is the hybridization term between actinide 5*f* states and states of the valence band – this band is filled up to the Fermi level ε_F_ which was set to zero in the calculations. The energy dependence of *V*(ε_υ_)^2^ over the valence band was assumed to have a semi-elliptical behavior, 

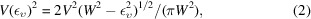

where *W* is the width of the valence band. *H*_multiplet_ represents the Coulomb, exchange and spin–orbit interactions for the actinide ion (Butorin *et al.*, 2016*b* ▸; Butorin, 2020 ▸, 2021 ▸).

To simplify calculations, the valence-band states were approximated by *N*_υ_ discrete energy levels which were equally spaced. The sufficient *N*_υ_ value was determined by convergence of the shape of calculated spectra.

To derive the HERFD-XAS spectra, the 3*d*–4*f* resonant inelastic X-ray scattering (RIXS) map for excitations across the U *M*_4_ absorption edge was calculated using the Kramers–Heisenberg formula, 

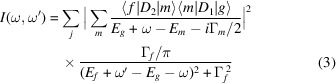

where |*g*〉, |*m*〉 and |*f*〉 are the ground, intermediate and final states of the spectroscopic process with energies *E*_*g*_, *E*_*m*_ and *E*_*f*_, respectively. ω and ω′ are the incident and emitted energies, respectively, and Γ_*m*_ and Γ_*f*_ are the lifetime broadenings of the RIXS intermediate and final states, in terms of half width at half-maximum (HWHM) of the Lorentzian function. Operators for optical dipole transitions *D* are expressed in terms of spherical tensor operators 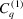 .

In the present RIXS calculations, the 90°-scattering geometry, which is usually applied in HERFD experiments, was taken into account. That leads (after setting the radial matrix elements *r* to unity) to the following dipole transition operators [see also a discussion by Butorin (2020 ▸)],

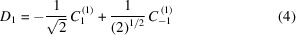

and 

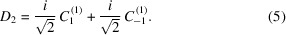

The HERFD-XAS spectrum is represented by a linear cut of such a 3*d*–4*f* RIXS map parallel to the incident energy axis at a constant emitted energy (the energy of the maximum of the *M*β X-ray emission line in this case) in the plane of emitted versus incident energies.

The conventional isotropic XAS spectra at the U *N*_4,5_ edges were calculated using the equation 

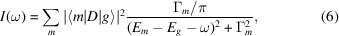

where |*g*〉 and |*m*〉 are the ground and XAS final (RIXS intermediate) states of the spectroscopic process with energies *E*_*g*_ and *E*_*m*_, respectively. *D* is the operator for the optical dipole transition with the incident photon energy represented by ω and lifetime broadening Γ_*m*_ of the XAS final (RIXS intermediate) state in terms of HWHM.

The 4*f* XPS spectrum was calculated using the following equation, 

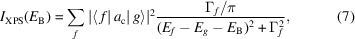

where |*g*〉 and |*f*〉 are the ground and XPS final states of the spectroscopic process with energies *E*_*g*_ and *E*_*f*_, respectively. *E*_B_ is the binding energy, and *a*_c_ is the annihilation operator of a core electron and Γ_*f*_ is a lifetime broadening of the final state in terms of HWHM.

The necessary Slater integrals, spin–orbit coupling constants and matrix elements were obtained with the *TT-MULTIPLETS* package which combines Cowan’s atomic multiplet program (Cowan, 1981 ▸) (based on the Hartree–Fock method with relativistic corrections) and Butler’s point-group program (Butler, 1981 ▸), which were modified by Thole *et al.* (1988 ▸), as well as the charge-transfer program written by Thole and Ogasawara.

To compare with the experimental data, it is usually necessary to uniformly shift the calculated spectra on the photon energy scale because it is difficult to reproduce very accurately the absolute energies in this type of calculation.

## 4. Results and discussion

**Figure 1:**
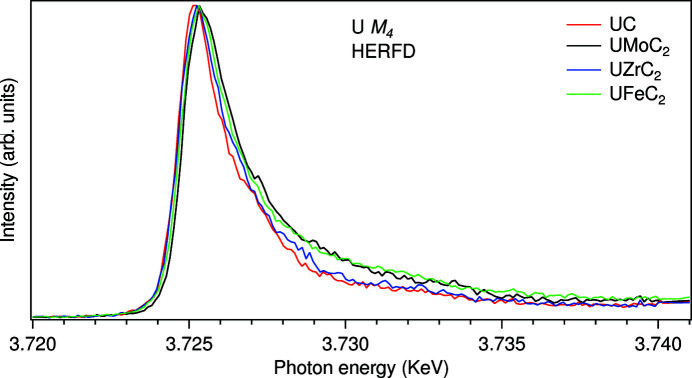
U *M*_4_ HERFD-XAS spectra of uranium carbides.

Fig. 1 ▸ displays the U *M*_4_ HERFD-XAS spectra of UC and UMeC_2_ (Me = Fe, Zr, Mo). The spectra appear to differ from each other. The spectra of UMeC_2_ reveal shifts to the high-energy side compared with those of UC. They also appear to be broader than those of UC due to an increased relative intensity on the high-energy side of the spectra. The values of observed shifts differ for UZrC_2_, UFeC_2_ and UMoC_2_. The largest shift of ∼0.25 eV is found for UMoC_2_ and the smallest (∼0.1 eV) for UZrC_2_. The shift for UFeC_2_ is close to that for UMoC_2_.

Overall, these shifts are much smaller than chemical shifts observed for uranium oxides. When going from the U^IV^ system, such as UO_2_ (Kvashnina *et al.*, 2013 ▸), to the U^V^ system, such as U_4_O_9_ (Kvashnina *et al.*, 2013 ▸) and NaUO_3_ (Butorin *et al.*, 2016*a* ▸), and further to the U^VI^ system, such as UO_3_ (Popa *et al.*, 2016 ▸; Butorin *et al.*, 2017 ▸), the chemical shift for the U *M*_4_ HERFD-XAS spectrum is as large as 1.0 eV.

**Figure 2:**
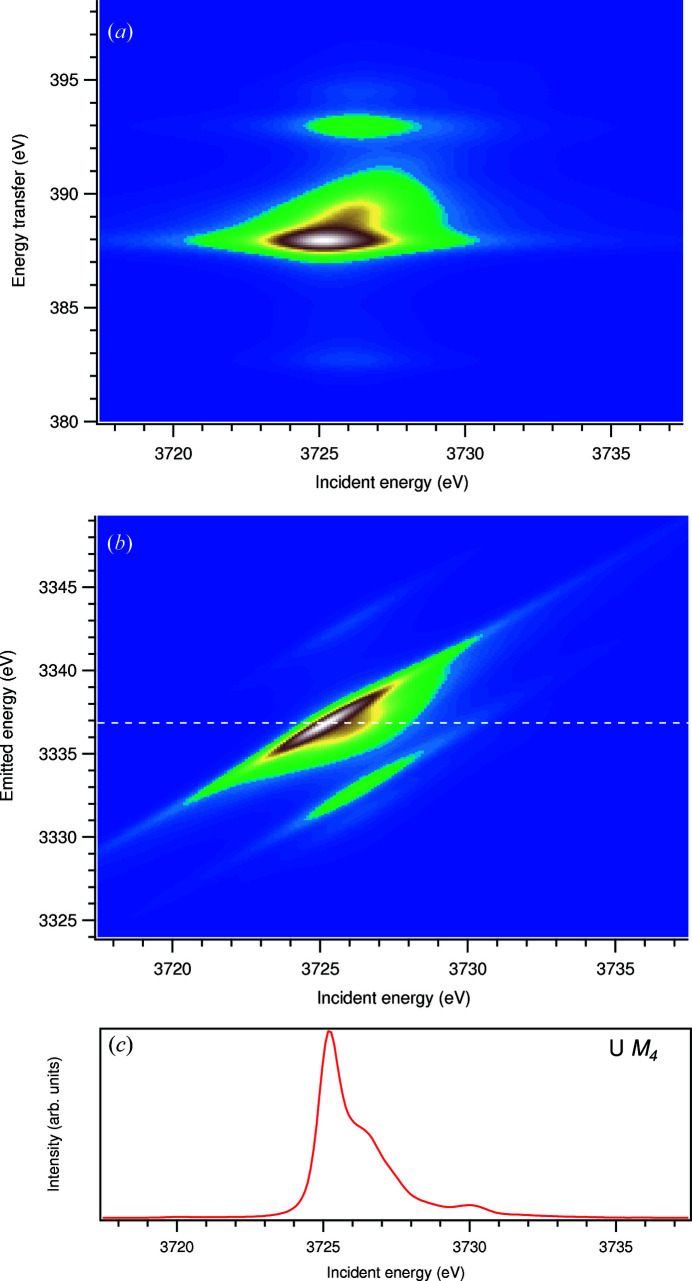
The 3*d*–4*f* RIXS map of UC with the incident energy on the *x* axis and the energy transfer (*a*) or emitted energy (*b*) on the *y* axis. The incident energy varies across the U *M*_4_ edge. Panel (*c*) shows a HERFD cut (red curve) of the 3*d*-to-4*f* RIXS map along the incident energy axis at an emitted energy corresponding to the RIXS maximum. This cut is indicated by a dashed line in (*b*).

In order to extract information about the ground state of UC, we calculated the core-to-core (3*d*–4*f*) RIXS map of UC for incident photon energies across the U *M*_4_ edge using the AIM approach (Fig. 2 ▸). The calculated U *M*_4_ HERFD-XAS spectrum of UC [Fig. 2 ▸(*c*)] is derived as a cut of this map through the maximum of the RIXS intensity at the constant emitted energy and along the incident energy axis. This cut is indicated by a white dashed line in Fig. 2 ▸(*b*).

In the AIM calculations, the ground, intermediate and final states of the system were each described by a linear combination of three electronic configurations: 5*f* ^2^, 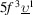 and 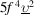 are in the ground state; 3*d* ^9^5*f* ^3^, 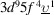 and 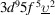 are in the intermediate state; 4*f* ^13^5*f* ^3^, 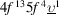 and 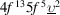 are in the final state. 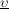 stands for an electronic hole in the valence-band states. The Slater integrals were reduced to 70% of their *ab initio* Hartree–Fock values to account for the configuration interaction effect (Lynch & Cowan, 1987 ▸).

In the limit of the hybridization term *V* → 0, the difference between the configuration averaged energies for the ground state can be written as 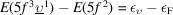 and 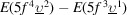 = 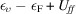 . For the intermediate (final) state, this difference is 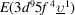 − 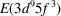 = 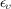 − 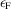 + 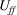 − 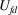 and 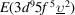 − 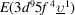 = 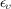 − 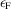 + 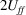 − 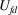 [ 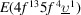 − 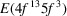 = 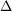 + 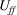 − 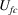 and 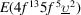 − 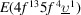 = 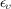 − 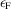 + 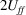 − 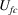 ]. Treated as parameters, the (ε_υ_ − ε_F_), *U*_*ff*_, *U*_*fd*_ and *U*_*fc*_ values were taken to be −0.5, 3.0, 3.5 and 3.5 eV, respectively. The hybridization term (hopping matrix element) between 5*f* ^2^ and 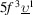 configurations and between 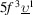 and 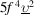 configurations in the ground state was taken as *V* = 0.6 eV. The same value of the *V* parameter was used for intermediate and final states of the RIXS process. The width of the valence band was taken to be *W* = 4.0 eV and *N*_υ_ = 8. Γ_*m*_ and Γ_*f*_ were set to 1.6 and 0.25 eV, respectively (Campbell & Papp, 2001 ▸).

At first glance, the RIXS intensity map in Fig. 2 ▸ shows a pattern which is somewhat different from that calculated for the U^IV^ ion (Butorin, 2020 ▸), *i.e.* for the 5*f* ^2^ ground-state configurations. For example, the structure at the energy transfer of ∼389.2 eV in Fig. 2 ▸(*a*), which corresponds to the structure at the incident energy of ∼3726.6 eV in the HERFD cut [Fig. 2 ▸(*c*)], shows a higher relative intensity than that for the 5*f* ^2^ case. Furthermore, the structures at the energy transfer of around 382.7, 388.0 and 393.0 eV appear to be broader than the corresponding structures for the 5*f* ^2^ case because of the U 5*f* hybridization with valence states and mixture of the 4*f* ^13^5*f* ^3^, 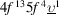 and 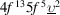 configurations. As in case of the 5*f* ^2^ (Butorin, 2020 ▸) configuration, the structure at the energy transfer ∼382.7 eV is a result of the 4*f*–5*f* exchange interaction in the final states of the spectroscopic process while the structure at the energy transfer ∼393.0 eV is rather a combination of the result of the 4*f*–5*f* interaction and a contribution from the charge-transfer satellite due to the U 5*f* hybridization with valence states. The latter structure is represented in the HERFD cut [Fig. 2 ▸(*c*)] by the satellite at an incident energy of ∼3730.5 eV, *i.e.* about 5.5 eV above the main line.

**Figure 3:**
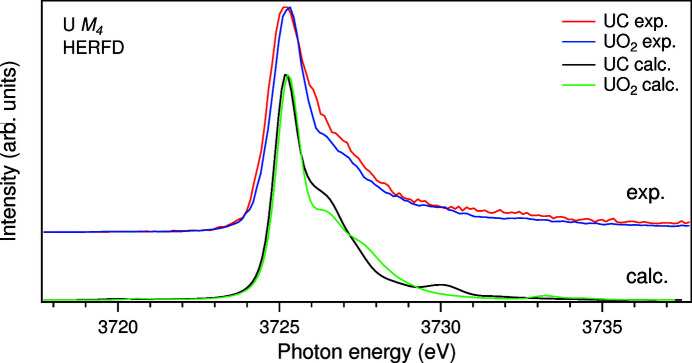
Calculated and measured U *M*_4_ HERFD-XAS spectra of UC and UO_2_.

The calculated U *M*_4_ HERFD-XAS spectrum of UC was found to be in agreement with the measured one (see a comparison in Fig. 3 ▸), thus supporting the choice of the model parameter values used in the AIM calculations. For these parameter values, the contributions of the 5*f* ^2^, 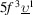 and 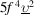 in the ground state were obtained to be 16%, 62% and 22%, respectively. This results in a 5*f* occupancy of *n*_*f*_ = 3.05 electrons. Due to the metallic character of UC and a negative value of ε_υ_ − ε_F_, the 5*f* occupancy significantly increases as a consequence of hybridization of the 5*f* states with the states of the valence band.

Fig. 3 ▸ also shows a comparison of the measured and calculated U *M*_4_ HERFD-XAS spectra of UO_2_. The calculated spectrum is a cut of the 3*d*–4*f* RIXS map through the maximum of the RIXS intensity which was obtained using the same AIM parameter values as used by Butorin *et al.* (2016*b* ▸) (the only difference is that in the present calculations all the Slater integrals were reduced to 80% of their Hartree–Fock values). In this case, the derived 5*f* occupancy in the ground state of UO_2_ is *n*_*f*_ = 2.24 electrons. The calculated and experimental U *M*_4_ HERFD-XAS spectra of UO_2_ are in a good agreement with each other. The calculations also reproduce the differences between the measured HERFD-XAS spectra of UC and UO_2_. In particular, the relative intensity of the high-energy shoulder at around 3726.7 eV is found to be lower for UO_2_, as in the experiment, although the intensity at ∼3728.0 eV is somewhat higher for UO_2_ than for UC, which is not observed experimentally. Note that the energy dependence of *V*(ε_υ_)^2^ over the valence band was approximated by a semi-elliptical function. The use of the function obtained with the DFT method should further improve agreement between calculations and experiment.

The AIM calculations reveal a significant deviation in 5*f* occupancy *n*_*f*_ from the values expected for the formal valency of U in UC. The obtained value of *n*_*f*_ in UC, which is 3.05 electrons, is significantly higher than 2.24 electrons in UO_2_. However, despite a large difference in the *n*_*f*_ and charge values between UC and UO_2_, the observed chemical shift between the recorded U *M*_4_ HERFD-XAS spectra of UC and UO_2_ is quite small (∼0.1 eV). Due to higher *n*_*f*_, a better shielding of the U nucleus in UC versus UO_2_ is expected, thus leading to a lower effective charge and consequently to a lower excitation energy for a core electron. On the other hand, the degree of the delocalization of the 5*f* states, which is another factor expected to influence the value of the chemical shift of the spectra, is significantly higher in UC, as compared with UO_2_. The delocalized 5*f* states respond much less to the attractive core-hole potential (as compared with the localized 5*f* states), thus reducing the chemical shift of the spectra to the low-energy side. Indeed, it has been shown for intermetallic systems that the chemical shifts of the HERFD-XAS spectra are significantly reduced (Kvashnina *et al.*, 2017 ▸).

Furthermore, due to the metallic character of UC, electron–hole pair excitations across the Fermi level are expected to exist in this carbide. It has been shown (Nakazawa & Kotani, 2002 ▸) that this type of excitation may be important for the RIXS process and since the HERFD profile at the U *M*_4_ edge is defined by the 3*d*–4*f* RIXS, such a dependence can influence the chemical shift of the HERFD-XAS spectra as well. It is also important to note that the 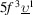 configuration which was estimated to provide the largest contribution to the ground state of UC is not the same as the pure 5*f* ^3^ configuration, especially if we consider their influence on the chemical shift of the spectra.

The AIM calculations for UMeC_2_ would require the introduction of a second set of model parameters to take into account the U 5*f*–Me 3*d* or 4*d* hybridization. This makes calculations much more complicated because of a significant increase in the number of states to be processed. Therefore, such calculations were left out of the scope of this paper. However, U 5*f*–Me 4*d*(3*d*) hybridization can indeed be a reason for an additional broadening of the U *M*_4_ HERFD-XAS spectra of UMeC_2_ as compared with UC (Jain *et al.*, 2013 ▸). Since the 4*d* band is expected to be wider than the 3*d* band, it is not surprising that the U *M*_4_ HERFD-XAS spectrum of UMoC_2_ is broader than that of UFeC_2_. The multi-electron excitations across the Fermi level to the U 5*f*–Me 4*d*(3*d*) hybridized states can be a reason for additional shifts of the U *M*_4_ HERFD-XAS spectra of metallic UMeC_2_ as compared with those of UC. As a whole, the observed small differences between UC and UMeC_2_ suggest that the UMeC_2_ materials have the predominant contribution of the 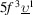 configuration in the ground state as well.

**Figure 4:**
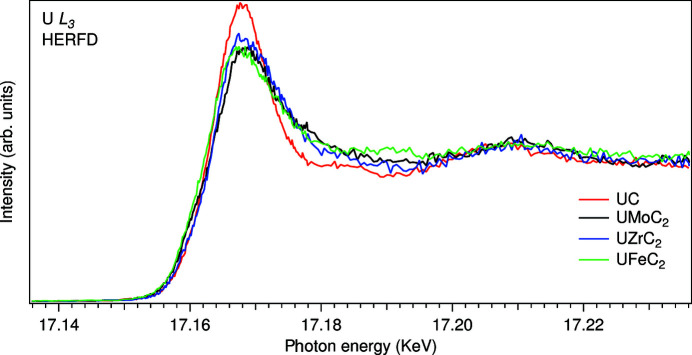
U *L*_3_ HERFD-XAS spectra of uranium carbides.

The differences between UC and UMeC_2_ are more emphasized in the U *L*_3_ HERFD-XAS spectra which probe the unoccupied U 6*d* density of states (DOS) in these compounds (Fig. 4 ▸). Due to a much higher degree of delocalization of the actinide 6*d* states as compared with actinide 5*f* states, the interpretation of the actinide *L*_3_ XAS spectra in terms of the probed actinide 6*d* DOS is more appropriate. The U *L*_3_ white line at around 17168 eV in the spectra of UMeC_2_ is much broader than that of UC due to a wider spread of contributing U 6*d* states in UMeC_2_ as a result of the U 6*d*–Me 4*d*(3*d*) hybridization. Particularly for UFeC_2_, the white line broadening on the low-energy side as compared with that of UC is also observed which can be explained by strong peaking of the unoccupied Fe 3*d* DOS close to the Fermi level (Jain *et al.*, 2013 ▸) and consequent hybridization of the U 6*d* states with Fe 3*d* states. The observed differences would be difficult to detect in conventional U *L*_3_ XAS spectra since the core-hole lifetime broadening (U 2*p*_3/2_) is around 4.0 eV (HWHM) (Campbell & Papp, 2001 ▸) versus ∼1.6 eV (U 3*d*) in the case of HERFD-XAS (Vitova *et al.*, 2010 ▸; Kvashnina *et al.*, 2014 ▸). Smaller core-hole lifetime broadening can be achieved with the XAS measurements at the U *N*_6,7_ edges, which also probe the U 6*d* states (Butorin *et al.*, 2016*c* ▸), but that requires the ultra-high-vacuum environment around the sample.

**Figure 5:**
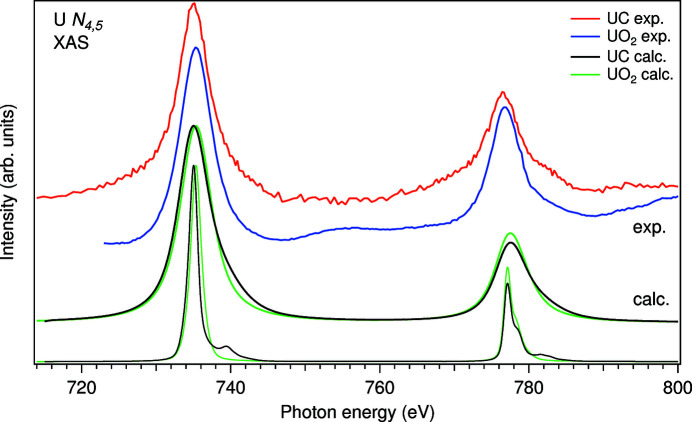
Calculated and measured U *N*_4,5_ XAS spectra of UC and UO_2_.

Fig. 5 ▸ displays the U *N*_4,5_ XAS spectrum of UC measured in the soft X-ray range, which is compared with that of UO_2_. The U *N*_4,5_ XAS spectrum of UO_2_ is the same as that published by Butorin *et al.* (2016*b* ▸). Fig. 5 ▸ also includes the results of AIM calculations for the U 4*d* edges of UC and UO_2_. Two sets of the calculated spectra with reduced and full 4*d* core-hole lifetime broadening Γ_*m*_ are shown. The experimentally determined core-hole lifetime broadening (∼2.1 HWHM) of the U 4*d* levels is quite large (Campbell & Papp, 2001 ▸), making the U *N*_4,5_ XAS spectrum appear as two structureless lines at ∼735.3 and ∼776.7 eV separated by the 4*d* spin–orbit interaction. Therefore, to emphasize the differences between UC and UO_2_ and to compare with the spectra at the U 3*d* edge, the reduced core-hole lifetime broadening (0.25 eV HWHM) was also used for the calculated U *N*_4,5_ XAS spectra. This value is the same as the one for the final state of the U 3*d*–4*f* RIXS process in the calculations of the U *M*_4_ HERFD-XAS spectra (see above).

For UO_2_, the U *N*_4,5_ XAS spectrum was calculated using the values for the AIM parameters from the work of Butorin *et al.* (2016*b* ▸). For UC, the AIM parameter values in the case of the U *N*_4,5_ XAS spectrum were the same as in the calculations of U 3*d*–4*f* RIXS and U *M*_4_ HERFD-XAS. Besides the same ground-state description, the final state of the U 4*d* XAS was represented by a linear combination of 4*d* ^9^5*f* ^3^, 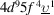 and 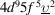 configurations. The observed differences between measured U *N*_4,5_ XAS spectra of UC and UO_2_ as well as between calculated spectra are similar to those in the case U *M*_4_ HERFD-XAS: the measured U *N*_4,5_ XAS lines of UC show some low-energy shift as compared with those of UO_2_ and the relative intensity of the shoulder at ∼779.5 eV in the calculated U *N*_4_ XAS spectrum of UC (see the spectrum with the reduced core-hole lifetime broadening in Fig. 5 ▸) is higher than that of UO_2_. Note that the broad structure at ∼753 eV in the U *N*_4,5_ XAS spectrum of UO_2_ represents transitions to the 7*p* continuum which are not taken into account in the calculations.

For 4*d* edges of actinides, the branching ratio of the *N*_5_ and *N*_4_ lines was argued to be a characteristic of the actinide oxidation state and 5*f* occupancy *n*_*f*_ (Moore & van der Laan, 2009 ▸). The branching ratio is derived as *I*_5/2_/(*I*_5/2_ + *I*_3/2_), where *I* is the integrated intensity of a line. It was demonstrated that a gradual decrease of the relative *N*_4_ intensity and a corresponding increase of the branching ratio occurs on going from the *n*_*f*_ = 1 system (Th metal) to the *n*_*f*_ = 6 system (Am metal) with reference to the oxidation state and the 5*f* occupancy. The measured U *N*_4,5_ XAS spectrum of UC (Fig. 5 ▸) indeed shows an increase in the branching ratio (0.69) as compared with that of UO_2_ (0.68), thus suggesting higher 5*f* occupancy in UC. That is also supported by the estimation of the branching ratio for the calculated U *N*_4,5_ XAS spectra which gives 0.70 versus 0.68 for UC and UO_2_, respectively.

**Figure 6:**
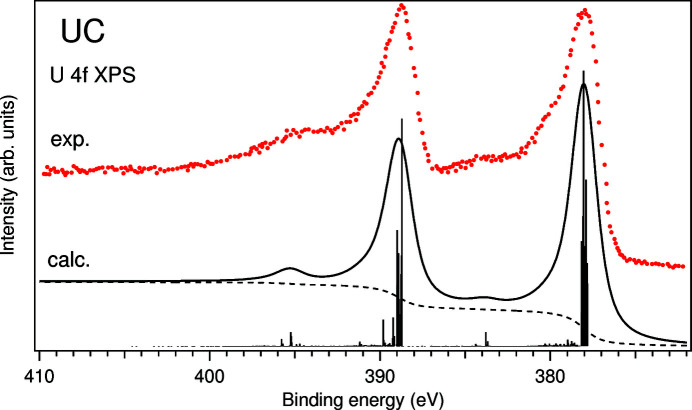
Calculated and measured (Ejima *et al.*, 1993 ▸) U 4*f* XPS spectra of UC.

To confirm the choice of the values of AIM parameters in the 3*d*–4*f* RIXS, U *M*_4_ HERFD-XAS and U *N*_4,5_ XAS calculations for UC, calculations of the U 4*f* XPS spectrum (Ejima *et al.*, 1993 ▸) of UC were also performed using the AIM approach. The same set of AIM parameter values as used for the 3*d*–4*f* RIXS, U *M*_4_ HERFD-XAS and U *N*_4,5_ XAS calculations was applied. The ground state was again described by a linear combination of the 5*f* ^2^, 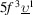 and 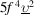 configurations, while a combination of the 4*f* ^13^5*f* ^2^, 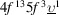 and 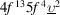 configurations was used for the final state of the spectroscopic process. The result of the calculations is compared with the measured spectrum (Ejima *et al.*, 1993 ▸) in Fig. 6 ▸. One can see that the relative intensity and energy separation of the satellites appearing at ∼6 eV above the 4*f*_7/2_ and 4*f*_5/2_ main lines on the binding energy scale are reproduced in the calculated spectrum. These satellites were initially assigned to the charge-transfer satellites (Ejima *et al.*, 1993 ▸) as a result of the 5*f* hybridization with the states in the valence band. However, based on the atomic multiplet calculations of the 4*f* XPS spectrum of the U^IV^ ion (Okada, 1999 ▸), it was pointed out that some structures may exist in the same energy range due to the 4*f*–5*f* interaction in the final state. Indeed, the results of our calculations suggest that ∼6 eV satellites are a product of both the 4*f*–5*f* interaction and charge-transfer effects. As a whole, the calculations of the U 4*f* XPS spectrum of UC confirm that the derived values of model parameters are appropriate for the description of the ground-state properties of UC.

## 5. Conclusion

The analysis of the high-energy spectroscopic data using the AIM approach suggests the predominant contribution of the 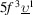 configuration in the ground state of UC. The 5*f* occupancy is estimated to be *n*_*f*_ = 3.05 electrons in this carbide. The introduction of 3*d* or 4*d* transition elements in uranium carbides, producing compounds such as UMeC_2_ (Me = Fe, Zr, Mo), does to some extent affect the chemical state of uranium in these systems as indicated by the shifts of the corresponding U *M*_4_ HERFD-XAS spectra. However, the small magnitude of the changes suggests that the ground state still has significant 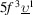 character.

## References

[bb1] Anderson, P. W. (1961). *Phys. Rev.* **124**, 41–53.

[bb2] Butler, P. H. (1981). *Point Group Symmetry Applications.* Boston, MA: Springer US.

[bb3] Butorin, S. M. (2020). *Inorg. Chem.* **59**, 16251–16264. 10.1021/acs.inorgchem.0c02032 PMC7672702 33136396

[bb4] Butorin, S. M. (2021). *J. Chem. Phys.* **155**, 164103. 10.1063/5.0062927 34717360

[bb5] Butorin, S. M., Kvashnina, K. O., Prieur, D., Rivenet, M. & Martin, P. M. (2017). *Chem. Commun.* **53**, 115–118. 10.1039/c6cc07684j 27878144

[bb6] Butorin, S. M., Kvashnina, K. O., Smith, A. L., Popa, K. & Martin, P. M. (2016*a*). *Chem. Eur. J.* **22**, 9693–9698. 10.1002/chem.201505091 27257782

[bb7] Butorin, S. M., Mancini, D. C., Guo, J.-H., Wassdahl, N., Nordgren, J., Nakazawa, M., Tanaka, S., Uozumi, T., Kotani, A., Ma, Y., Myano, K. E., Karlin, B. A. & Shuh, D. K. (1996). *Phys. Rev. Lett.* **77**, 574–577. 10.1103/PhysRevLett.77.574 10062845

[bb8] Butorin, S. M., Modin, A., Vegelius, J. R., Kvashnina, K. O. & Shuh, D. K. (2016*b*). *J. Phys. Chem. C*, **120**, 29397–29404.

[bb9] Butorin, S. M., Modin, A., Vegelius, J. R., Suzuki, M.-T., Oppeneer, P. M., Andersson, D. A. & Shuh, D. K. (2016*c*). *Anal. Chem.* **88**, 4169–4173. 10.1021/acs.analchem.5b04380 27008406

[bb10] Campbell, J. & Papp, T. (2001). *At. Data Nucl. Data Tables*, **77**, 1–56.

[bb11] Cowan, R. D. (1981). *The Theory of Atomic Structure and Spectra.* No. 3 in Los Alamos Series in Basic and Applied Sciences. Berkeley: University of California Press.

[bb12] Denecke, R., Väterlein, P., Bässler, M., Wassdahl, N., Butorin, S., Nilsson, A., Rubensson, J.-E., Nordgren, J., Mårtensson, N. & Nyholm, R. (1999). *J. Electron Spectrosc. Relat. Phenom.* **101–103**, 971–977.

[bb13] Ducher, R., Dubourg, R., Barrachin, M. & Pasturel, A. (2011). *Phys. Rev. B*, **83**, 104107.

[bb14] Eckle, M., Eloirdi, R., Gouder, T., Colarieti Tosti, M., Wastin, F. & Rebizant, J. (2004). *J. Nucl. Mater.* **334**, 1–8.

[bb15] Ejima, T., Murata, K., Suzuki, S., Takahashi, T., Sato, S., Kasuya, T., Ōnuki, Y., Yamagami, H., Hasegawa, A. & Ishii, T. (1993). *Physica B*, **186–188**, 77–79.

[bb16] Erbudak, M. & Keller, J. (1979). *Z. Phys. B*, **32**, 281–286.

[bb17] Ishii, T. (1993). *Physica B*, **186–188**, 21–25.

[bb18] Ito, T., Kumigashira, H., Takahashi, T., Yamamoto, E., Haga, Y. & Ōnuki, Y. (2001). *J. Magn. Magn. Mater.* **226–230**, 40–41.

[bb19] Jain, A., Ong, S. P., Hautier, G., Chen, W., Richards, W. D., Dacek, S., Cholia, S., Gunter, D., Skinner, D., Ceder, G. & Persson, K. A. (2013). *APL Mater.* **1**, 011002.

[bb20] Kvashnina, K., Kvashnin, Y. & Butorin, S. (2014). *J. Electron Spectrosc. Relat. Phenom.* **194**, 27–36.

[bb21] Kvashnina, K. O., Butorin, S. M., Martin, P. & Glatzel, P. (2013). *Phys. Rev. Lett.* **111**, 253002. 10.1103/PhysRevLett.111.253002 24483742

[bb22] Kvashnina, K. O., Walker, H. C., Magnani, N., Lander, G. H. & Caciuffo, R. (2017). *Phys. Rev. B*, **95**, 245103.

[bb23] Lynch, D. W. & Cowan, R. D. (1987). *Phys. Rev. B*, **36**, 9228–9233. 10.1103/physrevb.36.9228 9942789

[bb24] Moore, K. T. & van der Laan, G. (2009). *Rev. Mod. Phys.* **81**, 235–298.

[bb25] Nakazawa, M. & Kotani, A. (2002). *J. Phys. Soc. Jpn*, **71**, 2804–2814.

[bb26] Okada, K. (1999). *J. Phys. Soc. Jpn*, **68**, 752–755.

[bb27] Peniel, M., El Bekkachi, H., Tougait, O., Pasturel, M. & Noël, H. (2012). *Solid State Phenom.* **194**, 26–30.

[bb28] Popa, K., Prieur, D., Manara, D., Naji, M., Vigier, J.-F., Martin, P. M., Dieste Blanco, O., Scheinost, A. C., Prüßmann, T., Vitova, T., Raison, P. E., Somers, J. & Konings, R. J. M. (2016). *Dalton Trans.* **45**, 7847–7855. 10.1039/c6dt00735j 27063438

[bb29] Rodríguez-Carvajal, J. (2001). *IUCr Commission on Powder Diffraction, Newsletter*, **26**, 12–19.

[bb30] Shi, H., Zhang, P., Li, S.-S., Sun, B. & Wang, B. (2009). *Phys. Lett. A*, **373**, 3577–3581.

[bb31] Thole, B., Van Der Laan, G. & Butler, P. (1988). *Chem. Phys. Lett.* **149**, 295–299.

[bb32] Vigier, N., Den Auwer, C., Fillaux, C., Maslennikov, A., Noël, H., Roques, J., Shuh, D. K., Simoni, E., Tyliszczak, T. & Moisy, P. (2008). *Chem. Mater.* **20**, 3199–3204.

[bb33] Vitova, T., Kvashnina, K. O., Nocton, G., Sukharina, G., Denecke, M. A., Butorin, S. M., Mazzanti, M., Caciuffo, R., Soldatov, A., Behrends, T. & Geckeis, H. (2010). *Phys. Rev. B*, **82**, 235118.

[bb34] Wdowik, U. D., Piekarz, P., Legut, D. & Jagło, G. (2016). *Phys. Rev. B*, **94**, 054303.

[bb35] Wen, X.-D., Martin, R. L., Scuseria, G. E., Rudin, S. P. & Batista, E. R. (2013). *J. Phys. Chem. C*, **117**, 13122–13128.

[bb36] Yin, Q., Kutepov, A., Haule, K., Kotliar, G., Savrasov, S. Y. & Pickett, W. E. (2011). *Phys. Rev. B*, **84**, 195111.

[bb37] Zimina, A., Dardenne, K., Denecke, M. A., Doronkin, D. E., Huttel, E., Lichtenberg, H., Mangold, S., Pruessmann, T., Rothe, J., Spangenberg, T., Steininger, R., Vitova, T., Geckeis, H. & Grunwaldt, J.-D. (2017). *Rev. Sci. Instrum.* **88**, 113113. 10.1063/1.4999928 29195371

